# A School-Based Intervention Program to Reduce Weight Stigma in Adolescents

**DOI:** 10.3390/children12091208

**Published:** 2025-09-10

**Authors:** Mariacarolina Vacca, Silvia Cerolini, Anna Zegretti, Andrea Zagaria, Edoardo Mocini, Caterina Lombardo

**Affiliations:** 1 Department of Experimental Medicine, Sapienza University of Rome, 00185 Rome, Italy; 2 Department of Social Science, Guglielmo Marconi University, 00193 Rome, Italy; 3 Department of Psychology, Sapienza University of Rome, 00185 Rome, Italy; 4 Department of Systems Medicine, Tor Vergata University of Rome, 00133 Rome, Italy; 5 Department of Theoretical and Applied Sciences, eCampus University, 22100 Novedrate, Italy

**Keywords:** weight stigma, adolescents, secondary school, intervention

## Abstract

Background/Objectives: Weight-based stigma represents a pervasive psychosocial challenge affecting youth worldwide, with significant implications for mental and physical health. Although school-based interventions have been suggested as effective strategies to reduce weight bias, evidence regarding their content and efficacy in real-world educational contexts remains limited. The present study aimed to evaluate the effectiveness of a school-based intervention designed to reduce internalized weight stigma among Italian adolescents. Methods: A total of 539 secondary school students (Mage = 15.91 years; SD = 1.38; 51.5% males) from 10 schools in Italy were randomly assigned to either an intervention group (61.2%, *n* = 330) receiving the program or a minimal intervention control group (38.8%, *n* = 209). The intervention integrated psychoeducational modules and activities based on cognitive dissonance theory to address maladaptive weight-related attitudes and associated psychopathological outcomes. Pre- and post-intervention assessments evaluated internalized weight bias and body dissatisfaction in both groups. Results: A significant reduction in internalized weight bias was observed among students perceiving themselves as overweight following the intervention. Additionally, a marginally significant decrease in body dissatisfaction was reported in the intervention group from pre- to post-intervention. No comparable changes were found in the control group. Conclusions: These findings suggest that incorporating cognitive dissonance-based psychoeducational content within school-based programs may be a promising approach for reducing internalized weight stigma in adolescents. Further research is warranted to refine intervention strategies, explore the mechanisms underlying the observed effects, and assess the scalability and long-term impact of such initiatives in school settings.

## 1. Introduction

The past decade has seen a renewed importance in the concept of weight-based stigma [1], defined as negative weight-related attitudes toward people who present overweight (OW) or obesity (OB), expressed through negative stereotyping and/or discrimination, resulting in social denigration and prejudice [2,3]. Weight stigma is an overriding public health concern, particularly considering the steadily increasing prevalence of OB and OW and their health and psychological complications [4]. The worldwide prevalence rates of overweight and obesity amount to one-third of the global population, and current trends continue to rise dramatically [5], with an estimated reduction in life expectancy of 5–20 years depending on the severity of the condition [6].

In Italy, over ten percent of the adult population is affected by OB, and over one third presents OW [7]. Alarming rates of OW/OB have been observed in youth, more specifically among children [8] (30%) and adolescents (14.6%) [9], reaching the highest prevalence in Europe [10]. It has been reported that 40–50% of adults with OB/OW report perceiving negative weight-related biases, with 20% of them experiencing extremely high weight bias stigma [11]. Although obesity is associated with health risks, evidence indicates that individuals can maintain good health at higher body weights, highlighting the importance of body diversity and the need to reduce weight stigma [12].

Weight stigma may be particularly detrimental and treacherous when experienced during adolescence, a developmental period (i.e., 10–19 years) [13] accompanied by numerous body-related changes [14] and a contemporary emphasis on physical appearance [15]. Adolescence is also a critical period for identity development, during which body image and body satisfaction become closely linked to self-concept and social identity formation [16]. During this transitional period, physiological, social, and psychological changes make adolescents particularly vulnerable to developing a negative body image (i.e., an internalized view of one’s appearance) [17] and body dissatisfaction [18]. Friends, family, and peers are the most common sources of weight-based stigma in youth [19]. Adolescents are extremely vulnerable to weight-related judgment and stigma, especially from peers [20] and primarily in the school context [21]. The increasing awareness of physical appearances makes teenagers more prone to compare their bodies with those of peers and with the media’s portrayal of the “ideal” body [15,22]. Social media platforms can exacerbate weight stigma by increasing exposure to appearance-focused content and peer comparisons, potentially worsening body dissatisfaction and internalized stigma [23]. Youth anti-fat attitudes can result in different behavioral outcomes against victims, such as verbal teasing, bullying, and social exclusion [24]. Weight bias could also be negatively internalized in adolescents with OW or OB (i.e., weight self-stigma) [25] who attribute weight-based negative stereotypes to themselves, thus exacerbating the risk of social marginalization [26].

Recent estimates indicate that from 13 to 32% of adolescents report that they have been discriminated against by peers due to body weight [27]. The pernicious nature of weight stigma in youngsters has been supported by evidence suggesting that stigma exerts negative long-term effects on adolescent mental health (e.g., poorer social and emotional adjustment) [28]. Experiencing weight stigma is associated with maladaptive outcomes such as unhealthy eating, exercise and healthcare avoidance, and psychosocial difficulties including low self-esteem and social isolation [29,30,31]. Furthermore, weight stigma has been associated with adolescent anxiety, depression, low self-esteem, and overall poor health-related quality of life [28,32]. Longitudinal research has shown that weight-based teasing predicts dysfunctional eating and adverse weight outcomes after 15 years [3]. Evidence also demonstrated that using stigma as a weight-loss motivator is a societal misconception [33].

Understanding the widespread nature of weight stigma has encouraged innovative interventions aimed at reducing weight-related teasing among adolescents [34]. Intervention studies aimed at reducing weight stigma in youth populations remain limited and methodologically heterogeneous. Several studies have proposed education-focused interventions providing information about the multifactorial nature of obesity and its socio-cultural origins [35,36]. Other prejudice-reduction interventions promote acceptance and empathy in youngsters [37], based on the evidence that increased empathy discouraged stereotypes of stigmatized groups by reducing blame or controllability attributions and improving intergroup attitudes [38]. Results on the efficacy of weight bias intervention programs remain mixed. For example, in one eight-week prevention program with school-aged adolescents that included lectures related to weight bias, weight-related victimization (i.e., repeated exposure to negative actions or comments by others intended to harm an individual based on body weight) [39], and weight-biased attitudes significantly decreased from pre- to post-test [40]. Other studies using similar approaches did not detect changes in weight-based stereotypes [41]. Evidence suggests that school-based, multicomponent interventions incorporating psychoeducation, critical reflection on sociocultural body ideals, and promotion of inclusive peer climates may attenuate weight-biased attitudes among adolescents [20,42]. However, there is still a paucity of rigorously controlled, large-scale trials evaluating the real-world effectiveness of such programs within routine educational settings [43].

The present study was designed to address this gap by implementing and assessing an intervention targeting weight-based stigma among adolescents attending ten secondary schools in a metropolitan Italian context. The intervention comprised psychoeducational modules and cognitive dissonance-based activities, which are aimed at fostering critical awareness of weight-related stereotypes and promoting inclusive classroom norms. Importantly, the study employed a controlled design, administering pre- and post-intervention assessments to both intervention and minimal intervention control (MIC) groups using validated self-report measures. More specifically, different aspects related to weight stigma were examined. Adolescents who experience weight stigma often report heightened discrepancy between actual and ideal body image, resulting from the internalisation of societal thin-ideal standards [44]. Stigmatising experiences can exacerbate eating disturbances such as dietary restraint, binge eating, and compensatory behaviors, which may serve as attempts to conform to sociocultural body ideals or to alleviate weight-related distress [45]. Weight stigma can manifest in teasing and exclusionary behaviors toward peers with higher body weight, particularly in school contexts [46]. At the same time, adolescents experiencing internalized weight stigma are particularly vulnerable to weight-based victimization [29]

By simultaneously measuring weight stigma, eating disorder symptomatology, body dissatisfaction, and bullying involvement, this study adopts a comprehensive approach to evaluating the psychosocial outcomes of an anti-stigma intervention.

## 2. Materials and Methods

### 2.1. Participants

Adolescents were recruited from lower and upper secondary schools in the urban area of Rome. Schools were identified through prior collaborations with the research team and willingness to participate, ensuring representation of both urban and suburban contexts. Twelve schools were contacted by telephone and provided with a brief description of the project aims prior to being invited to participate in the study. Ten schools agreed to participate, and formal permission to conduct the research was obtained from school principals. Two weeks before data collection, parental consent forms and student assent forms were distributed to all students in the selected classrooms. Data collection was scheduled in agreement with individual teachers and was conducted only after written informed consent from both parents/legal caregivers and students was obtained. All the school classes were randomly assigned either to the intervention group, which participated in four structured sessions, or to a MIC that received only an introductory presentation of the project along with a brief session focused on raising awareness about weight stigma and weight-related bullying. The class-level randomization resulted in uneven group sizes (around 2:3), as the number of participants allocated in each condition was influenced by pre-existing differences in class enrollment.

To ensure participant anonymity, an alphanumeric code was assigned to each student for identification purposes prior to the administration of paper-and-pencil questionnaires, which took place during regular classroom hours. All students were invited to participate in the research without restrictions, and no compensation was provided for participation. The study was approved by the Institutional Review Board of the Department of Psychology at Sapienza University of Rome (date: 27 May 2022; prot number 0001069). The data for this study were collected with the support of a research grant from the Italian Department for Family Policies under the Presidency of the Council of Ministers.

### 2.2. Instruments

All students were asked to complete a series of self-reported questionnaires using their mobile phone/PC/tablet in group sessions with researchers on two occasions: before (T1) and after (T2) the intervention. The first assessment (T1) was used explore the cross-sectional relationships between a) weight stigma and aspects potentially implicated in negative attitudes toward individuals with and without OW/OB (as previously presented by Cerolini et al., 2024) [47], and between, b) bullying victimization and mental health outcomes (as previously presented by Vacca et al., 2024) [48]. During the second assessment (T2), the same set of questionnaires was administered again after the completion of the program to assess the intervention efficacy.

Socio-demographic information. Participants were required to indicate their age, sex, height, and weight.

Weight-related stigma and stereotypes. The Weight Bias Internalization Scale (WBIS) [49], in Italian adaptation form [50], was used for measuring the degree to which individuals accept and internalize negative weight-related stereotypes, attitudes, and commentary. The WBIS was specifically developed to assess the internalization of weight bias among individuals who perceive themself as being in a condition of overweight and obesity. Therefore, it was administered only to participants who classified themself as falling within the categories as reported in the first item (i.e., “Do you identify as a person with overweight or obesity?”). Participants responded to each item using a 7-point scale ranging from 1 (strongly disagree) to 7 (strongly agree). Higher scores indicate greater internalization of weight bias. Cronbach’s alpha in the present study was 0.904, showing excellent internal validity.

The Attitudes Toward Obese Persons Scale (ATOP) [51] in Italian adaptation form [52] was used to assess perceptions and attitudes about people with obesity. The scale consists of 16 items rated on a six-point Likert scale, ranging from −3 (strongly disagree) to 3 (strongly agree). Examples of proposed items include: “People with obesity should not expect to lead normal lives” or “People with obesity are generally sociable”. As stated by the original authors of the ATOP [51,52,53], lower scores indicated more negative attitudes. Cronbach’s alpha in the present sample was 0.765.

Eating Symptomatology. The Disordered Eating Questionnaire (DEQ) [54,55] evaluates eating difficulties and demonstrates high internal consistency. It consists of three sections: Section A includes 18 items focused on dysfunctional eating behaviors and four items concerning purging behaviors, rated on a 6-point frequency scale (0 = never; 6 = several times a day). Section B focuses on shape and weight concerns, rated on a 7-point Likert scale (0 = not at all; 7 = completely), and the last section (C) asks for the age, height, and weight of participants. All questions are presented concerning the period of the last three months. The questionnaire demonstrated good reliability in the validation sample (α = 0.90) [54]. Cronbach’s alpha in the present study was 0.935.

Body Dissatisfaction. The Contour Drawing Rating Scale (CDRS) [56] consists of nine gender-specific silhouettes with multiple body sizes ranging from the thinnest to the largest. Participants are asked to select the silhouette that best represents their current body size and the one that reflects their ideal body size. The discrepancy between these two choices was calculated, with negative ratings indicating more body dissatisfaction.

Bullying weight-related victimization. Two items from the revised Olweus Bully/Victim Questionnaire [57] adapted in the Italian context by Bacchini et al. [58] were used to assess physical appearance-based bullying perpetration (“I have teased someone about their physical appearance”) and victimization (“I have been teased about my physical appearance”) over the past six months. Responses were rated on a 5-point scale (1 = never; 2 = once/twice; 3 = 2/3 times a month; 4 = about once a week; 5 = several times a week), with higher scores indicating greater involvement in each role. The scale showed good internal consistency (ω = 0.848).

### 2.3. The Intervention Program

The intervention group participated in a six-session program, whereas the MIC group was exposed only to the program’s introductory phase—a brief, 20 min session aimed at raising awareness about weight stigma.

The first phase of the intervention delivered targeted training to the class group, focusing on the concept of weight stigma, the various forms of peer violence—including bullying and body shaming—and the recognition of at-risk behaviors. The sessions explored the multiple manifestations of peer violence and discriminatory conduct that threaten adolescents’ psychological, physical, moral, and social development, as well as their overall psychosocial well-being. Additionally, the training provided an overview of current national prevention, intervention, and victim protection frameworks, with particular attention to corresponding international and European systems, and guidelines for reporting harmful and discriminatory behaviors, especially those targeting children and adolescents.

The second phase, experiential in nature, engaged the class group in interactive workshops conducted through in-person sessions. These activities aimed to actively involve students in reflecting on the topics covered during the educational phase. Elements of the intervention drew on the principles of the validated Project Health protocol [42], which promotes healthy eating behaviors and active lifestyles. This approach encouraged participation in sports groups and the adoption of physically active routines. Concurrently, the program incorporated opportunities for peer bonding and group cohesion to cultivate an environment of inclusion and mutual acceptance.

The study team developed and implemented the intervention based on the biopsychosocial model of health [59], media literacy principles [60], and empirically supported strategies from the Project Health program [61]. The program comprised a total of six sessions, including two for pre- and post-assessment and four dedicated to the intervention (see below for a detailed description). Each lasting one hour per class, scheduled within school hours as either curricular or extracurricular activities, complemented by workshops and discussion groups. The number and length of sessions were adapted from existing evidence-based protocols (e.g., Project Health [42]) and balanced with the constraints of school timetables to optimise feasibility. Each session was facilitated by licensed psychologists. Regular supervisory meetings were conducted to ensure consistency and procedural fidelity across the sessions. A detailed preparatory training on the intervention framework was provided to facilitators, who also attended regular supervisory meetings to harmonise procedures and ensure consistency. Given the pilot and feasibility-oriented nature of the intervention, no formal manual or adherence checklists were employed. Fidelity was supported using detailed session scripts developed by the study team and delivered uniformly across classes.

Session 1: The initial session focused on introducing the concept of weight stigma, elucidating its definition, consequences, and the cyclical nature of stigmatization. Participants explored how stigma operates and its impact on individuals’ psychological and social well-being.

Session 2: The second session addressed the behavioral components associated with weight stigma, including bullying and discrimination. Key contributing factors such as familial influences, social environments, and body image concerns were discussed in depth to understand their role in exacerbating stigmatising behaviors.

Session 3: The third session was dedicated to cognitive restructuring exercises aimed at challenging maladaptive thought patterns. Topics included cognitive avoidance strategies, practical advice to resist unrealistic beauty ideals, and engaging in cognitive-behavioral therapy (CBT) inspired strategies to challenge difficult situations.

Session 4: Four common myths related to eating habits, body image, obesity, and stigma were debunked, and students were encouraged to adopt healthy behaviors. A set of short educational video modules was developed and delivered as a final part of the intervention protocol. The modules were designed to provide participants with evidence-based insights into the complex, multifactorial nature of obesity, emphasizing its recognition as a chronic disease rather than a result of individual failure. Topics included the biopsychosocial model of health [62], the role of environmental and genetic factors in obesity, the impact of weight stigma on treatment outcomes, the influence of diet culture and the commercial diet industry, as well as the dismantling of common nutrition myths. This session was conducted from a weight-neutral health perspective, emphasizing that good health can be achieved across a range of body sizes when individuals engage in recommended nutrition and physical activity behaviors [12].

At the end of the sessions, in order to sum up all the information learned and be able to apply it in practice, participants were asked to write a supportive letter to a friend struggling with body image/weight-related concerns, thus fostering empathy and self-reflection.

**Figure 1:**
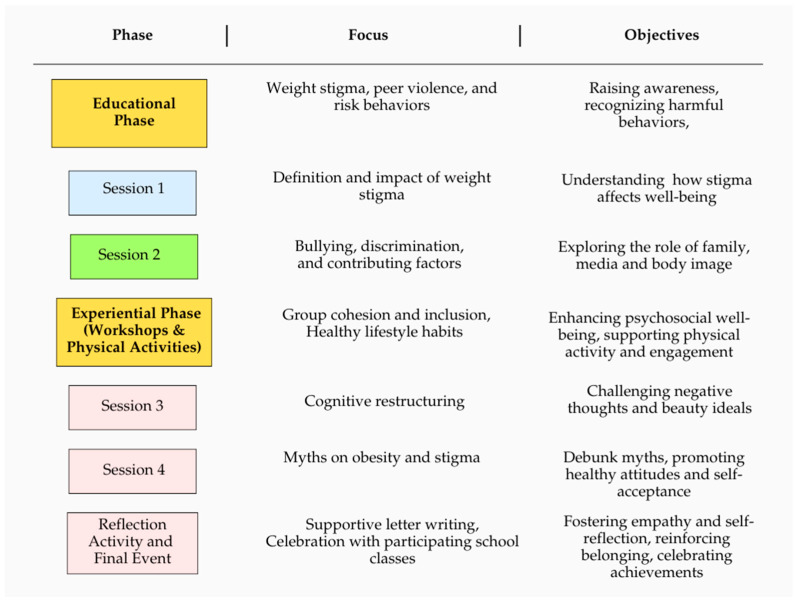
The Structured Intervention Program.

At the end of the intervention phase and at the fulfilment of the second assessment, a celebratory event was planned for all the school divisions involved in the study. The celebration event was not part of the experimental content, and all the students from both the groups (intervention vs. MIC) participated. In collaboration with physical education teachers, recreational and team sports activities (e.g., tournaments) were organized to further support physical activity and social engagement. A t-shirt and booklet were distributed to all participants as the project’s gadgets, including the slogans that some of the participating students had created to support the project’s initiatives (e.g., “Stop body shaming!”, “All the bodies are beautiful!”, etc.). Particular attention was dedicated to the collection and distribution of the t-shirts (all sizes were available, making sure that everyone took their own independently and privately, see Figure 1 for a description of the program activities).

**Figure 2:**
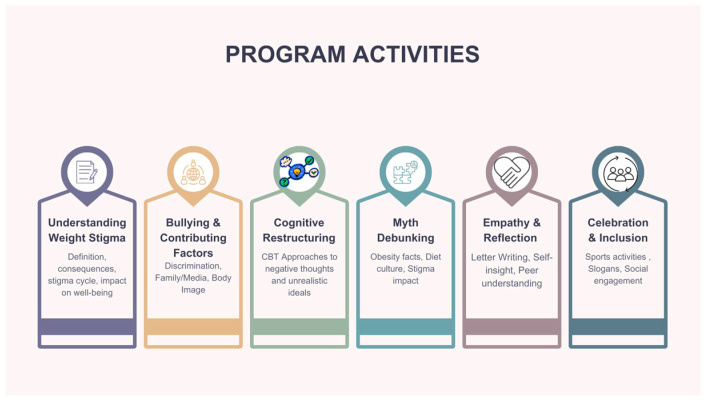
Overview of Proposed Intervention Activities.

Students were also provided with an emergency support service for weight-based bullying, consisting of a no-cost helpline, available two days per week and operated in collaboration with a national child protection organization—Fondazione S.O.S Il Telefono Azzurro ETS—and a designated email address coordinated by the project team. This approach enabled continuous support throughout the intervention, ensuring that participants were not left unaided and were facilitated to disclose personal concerns or difficulties related to weight stigma and victimization (see Figure 2 for an overview of the proposed intervention). 

### 2.4. The Psychoeducation Program for the MIC Group

School classes that were randomized in the MIC group received a 20 min psychoeducational session focused on the definition of weight stigma and its potential consequences on psychological well-being. This constituted a brief component of the initial intervention session administered to the intervention group and was implemented immediately before the pretest (T1). Students were encouraged to seek additional information, and they had continuous access to the free helpline throughout the duration of the program at the schools.

### 2.5. Data Analysis

Data were analysed using Jamovi v. 2.6.26 (https://www.jamovi.org/, accessed on 12 May 2025).

Firstly, socio-demographic descriptive statistics were calculated to characterise the sample, and bivariate correlations among the main study variables were calculated at baseline (T1).

Afterwards, four separate two-way mixed-design ANOVAs were performed to examine the effects of the intervention on WBIS, DEQ, ATOP, and CDRS scores, by considering Group (intervention vs. MIC) as the between-subjects factor and Moment of assessment (pre vs. post intervention) as the within-subjects factor. In the event of a significant Group × Moment interaction, analyses of simple main effects were planned to explore changes over time within each group separately.

To further explore the efficacy of the intervention and assess potential moderating effects, a series of exploratory ANCOVAs were conducted. For each outcome (WBIS, DEQ, ATOP, and CDRS), the post-treatment score (T2) was entered as the dependent variable, with Group (intervention vs. MIC) as the between-subjects factor and the corresponding baseline score (T1) as a covariate. In addition, BMI and sex were included as covariates, and their interactions with Group were tested to examine potential moderating effects. This approach allowed us to assess whether the intervention effect was consistent across BMI and sex, and to control for potential baseline differences in the outcomes.

Effect sizes were expressed using partial eta squared (ηp^2^), with values of 0.01, 0.06, and 0.14 denoting small, moderate, and large effects, respectively [63]. The α level was set at 0.05 to determine statistical significance.

## 3. Results

### 3.1. Socio-Demographic Characteristics

A total of 539 adolescents completed the pre-intervention assessment and were enrolled in the study. Of these, 38.8% (*n* = 209) were in the MIC condition and 61.2% (*n* = 330) in the intervention condition. Participants had a mean age of 15.91 years (SD = 1.38; range: 13–21) and were sex-balanced (51.5% males and 48.5% females). Of the total sample, 21.89% (comparison group: *n* = 53; experimental group: *n* = 65) reported having teased someone about their weight or physical appearance at least once in the past six months, while 22% (comparison group: *n* = 44; experimental group: *n* = 75) reported having personally experienced teasing for the same reasons during the same period. Notably, 25.2% of the sample reported having experienced body shaming by family members, while 26.8% reported experiencing body shaming by peers.

Moreover, a total of 73 adolescents self-identified in a condition of overweight or obesity (Mean BMI = 26.49 kg/m^2^, SD = 4.04) and completed the WBIS. This subgroup had a mean age of 15.81 years (SD = 1.31; range: 14–19) and was predominantly female (65.8%). Critically, 53.4% of these participants reported having experienced body shaming by family members, while 57.5% reported experiencing body shaming by peers. Of this subgroup, 31.5% (*n* = 23) were assigned to the MIC condition and 68.5% (*n* = 50) to the intervention condition.

Detailed descriptive statistics of the investigated variables are summarised in Table 1.

### 3.2. Bivariate Correlations

Pearson’s correlation coefficients representing bivariate correlations among variables measured at baseline (T1) are summarized in Table 2. More specifically, DEQ scores were strongly correlated with WBIS (r = 0.700, *p* < 0.001) and CDRS (r = −0.610, *p* < 0.001) scores. Moreover, WBIS scores were significantly and moderately correlated with CDRS scores (r = −0.373, *p* < 0.001).

### 3.3. Two-Way Mixed-Design ANOVAs

Two-way mixed-design ANOVAs were implemented with Group (intervention vs. MIC) as the between-subjects factor and Moment (pre vs. post intervention) as the within-subjects factor. Four distinct analyses were conducted, considering WBIS, DEQ, ATOP, and CDRS scores as dependent variables, respectively. Detailed results are reported in Supplementary Table S1.

#### 3.3.1. Attitudes Toward Obese Persons Scores

ANOVA revealed no significant Group × Moment interaction, F(1, 494) = 0.076, *p* = 0.783, ηp^2^ = 0.000, indicating that the trajectory of ATOP scores from pre- to post-intervention did not differ between groups. Similarly, no significant main effects emerged for Moment (F(1, 494) = 0.005, *p* = 0.945, ηp^2^ = 0.000) or Group (F(1, 494) = 0.001, *p* = 0.973, ηp^2^ = 0.000), suggesting that neither time nor condition influenced participants’ attitudes toward obese persons.

#### 3.3.2. Weight Bias Internalization Scale Scores

Findings revealed no significant Group × Moment interaction, F(1, 71) = 0.126, *p* = 0.723, ηp^2^ = 0.002, indicating that changes in WBIS scores over time did not differ significantly between the two experimental conditions. Nonetheless, significant main effects of Group (F(1, 71) = 4.627, *p* = 0.035, ηp^2^ = 0.061) and Moment (F(1, 71) = 7.518, *p* = 0.008, ηp^2^ = 0.096) were observed, suggesting that, on average, participants in the intervention and MIC groups differed in their levels of internalized weight bias, and that WBIS scores changed significantly from pre- to post-intervention, regardless of group membership.

**Figure 3:**
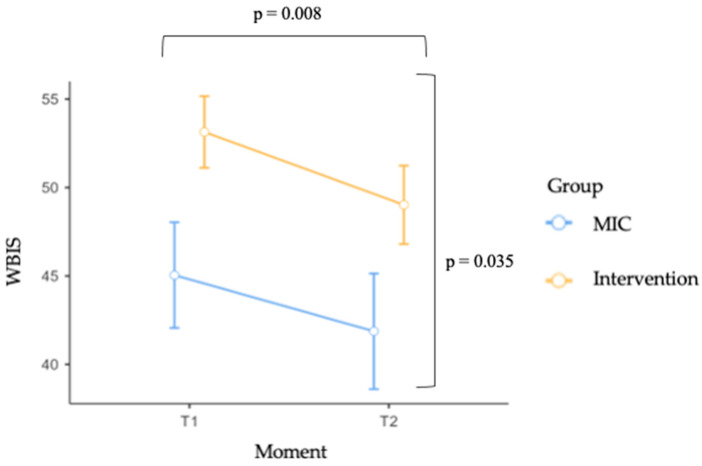
WBIS scores by group (MIC vs. Intervention) across time (Moment). Error bars represent standard errors of the mean (SE). Square brackets indicate the main effects of Group and Time.

To further explore the significant main effects, descriptive statistics were examined. On average, participants in the intervention group reported higher WBIS scores (M = 51.08, SE = 1.98) compared to those in the MIC group (M = 43.45, SE = 2.93), across both time points. Furthermore, WBIS scores decreased from pre (M = 49.09, SE = 1.80) to post (M = 45.44, SE = 1.97) intervention, suggesting that participants experienced a reduction in internalized weight bias over time, regardless of whether they were in the intervention or MIC condition. These patterns are graphically illustrated in Figure 3.

#### 3.3.3. Disordered Eating Questionnaire Scores

Findings revealed no significant Group × Moment interaction, F(1, 473) = 0.025, *p* = 0.875, ηp^2^ = 0.000, indicating that changes in DEQ scores over time did not differ between the two experimental conditions. Moreover, no significant main effects of Moment (F(1, 473) = 0.324, *p* = 0.569, ηp^2^ = 0.001) or Group (F(1, 473) = 1.193, *p* = 0.275, ηp^2^ = 0.003) were found.

#### 3.3.4. Contour Drawing Rating Scale Scores

**Figure 4:**
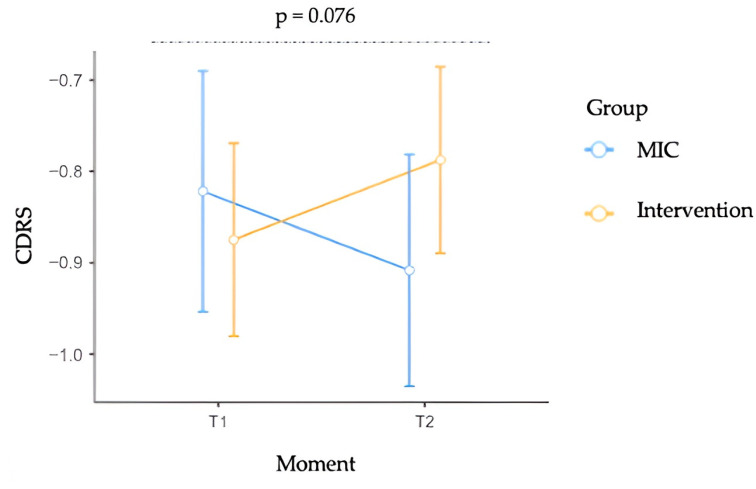
Contour Drawing Rating Scale (CDRS) scores by group (MIC vs. Intervention) across time (Moment). Error bars represent standard errors of the mean (SE). The dashed line indicates the Group × Moment interaction.

Interestingly, the analysis conducted on CDRS scores revealed a marginally significant Group × Moment interaction, F(1, 470) = 3.152, *p* = 0.076, ηp^2^ = 0.007, indicating that changes in body size dissatisfaction over time marginally differ between the MIC and intervention groups. Furthermore, no significant main effects of Moment (F(1, 470) = 0.001, *p* = 0.995, ηp^2^ = 0.000) or Group (F(1, 470) = 0.046, *p* = 0.831, ηp^2^ = 0.000) were found. The interaction effect is illustrated in Figure 4. The effect in the intervention group did not attain statistical significance (*p* > 0.05), yet a consistent direction of change was apparent, with the discrepancy between actual and desired body size decreasing from pre-intervention (M = −0.875, SE = 0.106) to post-intervention (M = −0.787, SE = 0.102).

### 3.4. Exploratory ANCOVAs

To further explore intervention efficacy while accounting for baseline differences, a series of ANCOVAs were conducted. For each outcome measure (WBIS, DEQ, ATOP, and CDRS), the post-treatment score (T2) was entered as the dependent variable, with Group (intervention vs. MIC) as the between-subjects factor. Baseline scores (T1), BMI, and sex were included as covariates. Interaction terms (Group × BMI and Group × Sex) were also included to examine their potential moderating effects.

#### 3.4.1. Attitudes Toward Obese Persons Scores

ANCOVA revealed no significant Group × Sex interaction for ATOP scores at T2, F(1, 473) = 3.309, *p* = 0.070, ηp^2^ = 0.007, suggesting that the effect of the intervention did not significantly differ by sex. Similarly, the Group × BMI interaction was not significant (F(1, 473) = 1.170, *p* = 0.280, ηp^2^ = 0.002). The main effect of Group also remained non-significant, F(1, 473) = 0.148, *p* = 0.701, ηp^2^ = 0.000, indicating that, after controlling for baseline scores, BMI, and sex, the intervention did not yield a significant change in ATOP scores compared to the MIC group.

#### 3.4.2. Weight Bias Internalisation Scale Scores

ANCOVA showed no significant Group × Sex interaction for WBIS scores at T2, F(1, 63) = 0.419, *p* = 0.520, ηp^2^ = 0.007, indicating that the intervention effect did not differ significantly between sexes. The interaction between Group and BMI was also non-significant (F(1, 63) = 0.079, *p* = 0.779, ηp^2^ = 0.001). Similarly, the main effect of Group was meaningless, F(1, 63) = 0.207, *p* = 0.651, ηp^2^ = 0.003, suggesting that, after accounting for baseline scores, BMI, and sex, the intervention had no significant impact on post-intervention WBIS scores compared to the MIC condition.

#### 3.4.3. Disordered Eating Questionnaire Scores

ANCOVA did not reveal a significant Group × Sex interaction on DEQ scores at T2, F(1, 452) = 0.828, *p* = 0.363, ηp^2^ = 0.002, suggesting that the intervention effect was not moderated by sex. Likewise, no significant interaction emerged between Group and BMI (F(1, 452) = 0.205, *p* = 0.651, ηp^2^ = 0.000). The main effect of Group was also negligible, F(1, 452) = 0.018, *p* = 0.893, ηp^2^ = 0.000, indicating that, when controlling for baseline scores, BMI, and sex, the intervention exerted no influence on post-intervention DEQ scores compared to the MIC condition.

#### 3.4.4. Contour Drawing Rating Scale Scores

ANCOVA did not reveal a significant Group × Sex interaction on CDRS scores at T2, F(1, 450) = 0.001, *p* = 0.996, ηp^2^ = 0.000, suggesting that the effect of the intervention did not differ by sex. Similarly, the Group × BMI interaction was not significant (F(1, 450) = 0.249, *p* = 0.618, ηp^2^ = 0.001). Importantly, the main effect of Group approached statistical significance, F(1, 450) = 3.831, *p* = 0.051, ηp^2^ = 0.008. Estimated marginal means showed that, after adjusting for baseline scores, BMI, and sex, participants in the intervention group reported a smaller discrepancy between actual and desired body size at post-intervention (M = −0.727, SE = 0.058) compared to those in the MIC group (M = −0.905, SE = 0.070).

## 4. Discussion

The findings of this study offer valuable insights into both the potential and limitations of a brief intervention aimed at reducing weight stigma and related aspects in high school students. Firstly, results highlight that 13.54% (*n* = 72) of the total sample self-identified as being in a condition of OB/OW. Importantly, more than half of these participants reported having experienced body shaming by family members and peers. This evidence aligns well with previous research highlighting the potential role of self-weight perception in influencing adolescents’ susceptibility to weight-based victimisations and suggesting that intervention efforts should address not only actual body weight, but also self-perceptions and concerns related to body image. Moreover, this finding confirmed previous evidence on the prevalence of weight bias among adolescents [64], especially in educational environments [65,66], and established school context as a fertile ground for the development of weight-based discrimination. Additionally, descriptive results suggest that more than 20% of the total sample reported having perpetrated or experienced appearance-based bullying. These findings confirm the pervasive nature of appearance and weight-based bullying–either acted as perpetrator or victim-among adolescents (e.g., [67]) and highlight the need for interventions designed to address both the victimization and the perpetration of these forms of bullying. Previous studies reported that between 11% and 54% of Italian school students experienced teasing related to physical appearance, with higher rates observed among students with more severe obesity [67]. Future research should investigate whether the effects of the intervention vary across students with different weight profiles. While appearance-related bullying is common, other forms of bullying (e.g., based on race, ethnicity, religion, sexual orientation) tend to have lower prevalence rates, generally ranging between 5% and 15% in adolescent populations [68,69]. This comparison highlights that, although all forms of bullying are problematic, appearance and weight remain among the most frequent triggers for peer victimisation.

This is true especially considering the developmental period of adolescence, characterised by profound physical, psychological, and social changes [70]. During this transitional period, a series of developmental processes occur, including increases in self-consciousness and awareness of external and sociocultural expectations [71]. Given the heightened relevance of peer approval and social conformity during adolescence, this population can be distinctively susceptible to questioning their self-worth and thus may develop weight bias in response to external sociocultural pressures [30,44]. Indeed, adolescents are particularly susceptible to weight stigma and its harmful effects, such as teasing, bullying, social isolation, physical health issues, and emotional distress [30]. One of the main contributors to weight stigma during adolescence is social media, which is widely used by teens around the world. These platforms often expose users to weight-related messages that tend to marginalise individuals with higher body weights, fostering the internalisation of weight bias [72]. For adolescents, experiencing weight stigma has been linked to higher rates of depression, anxiety, suicidal thoughts, disordered eating behaviors, and substance use [32]. Future works are needed to investigate potential risk factors and their reciprocal relationship influencing the structuration of weight-based stereotypes in youth, as well as potential health-related consequences.

Emerging evidence indicates that the phenomenon is also quite widespread in Italy, and experiences of weight stigmatisation, bullying, and body shaming are frequently reported in association with poorer health outcomes [47,48,67]. Our school-based intervention program was designed to reduce weight stigma and weight-related teasing through the face-to-face delivery of a four-session training, conducted by researchers who were all licensed psychologists or psychotherapists with expertise in this area.

The evaluation of our intervention program was primarily focused on the comparison between pre- and post-intervention scores of internalized weight stigma, alongside associated aspects including body dissatisfaction, eating symptomatology, and weight-based attitudes. Overall, no statistically significant effects of the intervention emerged for the majority of aspects considered, and the results should be interpreted with caution. The intervention yielded small effect sizes across the analyses, suggesting that the proposed program produced limited and heterogeneous changes rather than robust, sustained reductions in weight stigma and related factors.

More specifically, no significant intervention effects were found for explicit attitudes towards obesity (i.e., ATOP). Previous evidence emphasises a small to medium effect of existing interventions on explicit weight-biased attitudes and beliefs [73], implying that short-term interventions may be insufficient to alter these aspects in adolescents. However, in the subgroup of participants perceiving themselves as being in an overweight condition, a significant effect of Time occurred in predicting a reduction in internalized weight stigma (i.e., WBIS). The absence of a Group × Moment effect indicated similar changes in both groups—MIC and experimental—over time. Multiple explanations could be mentioned for this result. First, the low-intensity psychoeducation provided to the MIC group at the pre- and post-assessment may have been beneficial, suggesting that even a minimal education exposure could exert effects in reducing weight bias internalisation, similarly to a complete intervention protocol [29]. Second, the educators of students in both conditions were recommended to pay attention to weight stigma topics, in line with the intervention protocol, as suggested elsewhere [66]. This may have fostered the reduction in weight stigma across all the students involved. Future studies are needed to assess the impacts of teachers’ weight-related attitudes on students’ own perceptions and attitudes toward weight. Moreover, the main significant contribution of the Group indicated that internalized weight bias was generally higher in the intervention group, irrespective of the time point, suggesting that participants in the experimental condition may have exhibited a higher baseline level of weight bias, a trajectory which was maintained in post-intervention scores. This could reflect a “baseline balance effect” [74] and should be addressed as one limitation of this study. Furthermore, the sole contribution of time in predicting weight bias reduction in both groups may agree with the “Hawthorne effect”, a non-specific treatment effect where the awareness of being assessed may alter the participants’ responses and behaviors [75].

Results on body dissatisfaction revealed a marginally significant Group × Moment interaction effect, indicating that changes in this aspect over time marginally differ between the MIC and intervention groups. The effect did not reach statistical significance, and therefore must be interpreted cautiously, although a small reduction in the discrepancy between actual and desired body size was observed from pre- to post-intervention in the experimental group. This modest change aligns with previous observations [29] but should be interpreted with caution. While this finding, albeit limited, could encourage the potential value of integrating weight stigma-focused content in existing intervention protocols designed to improve body image concerns, alternative explanations of these effects—such as short intervention duration or sample characteristics—cannot be excluded. Considering the role of overvaluation of shape and weight as a core psychopathological determinant of eating disorders [76,77], further research is needed to determine whether more intensive programs could yield meaningful improvements.

No significant intervention effects were found for eating symptomatology (i.e., DEQ). Previous evidence of substantial improvement in eating disturbances after a weight-stigma focused intervention suggests heterogeneous results [78]. One potential explanation of this negligible effect could be the brief and limited duration of the present program, together with the fact that most weight bias intervention protocols mainly focus on attitudes rather than emotional and behavioral mechanisms underpinning eating symptomatology [78]. Future experimental studies should address this limitation by integrating these aspects into the intervention protocols.

This study clearly presented some limitations. First, the mere employment of self-reported measures may have constrained the robustness of the assessment, potentially enhancing the desirability bias. Future studies should consider integrating objective measures, such as the implicit association test (IAT) for weight bias [79], an experimental tool that captures automatic negative weight-based attitudes. Second, the inclusion of Italian secondary school students may have influenced the generalizability of findings due to cultural or educational contexts. For example, the Italian school system is characterised by teacher-led classroom activities, which may have supported the delivery of psychoeducational modules. Third, the heterogeneity of participant baseline weight bias levels limited the robustness of conclusions on intervention efficacy as well as their internal validity. Further research could employ blocking or stratification based on key baseline characteristics (e.g., weight stigma levels) to improve group balance. Fourth, randomisation was performed at the class rather than the individual level; the number of participants allocated to each condition was determined by pre-existing differences in class enrollment, leading to uneven group sizes. Moreover, a contamination of information may have taken place from the intervention group to the MIC group, due to potential informal communication between students across classes, which also may have diluted between-group differences. Future studies should address this issue by adopting a cluster-randomised design at the school level, thereby minimising the likelihood of cross-group information exchange. Sixth, since the control group received minimal psychoeducation on weight stigma, a true control condition was not established. Future studies could include an active control group with content unrelated to weight stigma or a waitlist control group to further strengthen internal validity. Furthermore, the absence of systematic attendance records limited our ability to examine potential dose–response effects of the intervention, which may have influenced the magnitude of the observed outcomes. Future studies should assess this aspect by recording adherence to the intervention sessions for each participant. Finally, the lack of a follow-up assessment hinders the evaluation of the intervention in the long term and the maintenance of the short-term effects over time. Moreover, this prevented the observation of potential score regression at follow-up in the MIC group. Future studies should address this point by including more occasions of post-intervention assessment. Finally, future studies should enhance intervention fidelity by employing standardised manuals and formal adherence checklists in addition to facilitator training and supervision.

Notwithstanding these limitations, the present study possesses the distinct strength of providing a unique and significant contribution to the scientific literature on weight stigma interventions, offering valuable recommendations for the development of future prevention and experimental protocols for a reduction in weight stigma and related aspects in educational settings. The principal strength of this intervention resides in its grounding on the international recommendation of the World Obesity Federation to reduce weight stigma (for a detailed description, see Nutter et al. [80]). In line with the position paper, our program tries to change the common social narratives, language, and images used to discuss body weight, obesity, and appearance and specifically: (1) uses non-stigmatizing language and imagery; (2) uses person-first language (describing what a person “has” rather than asserting what a person “is”); (3) engages in weight-neutral health promotion, especially in the sections targeting physical activity and nutritional aspect (e.g., the set of short educational video modules which focuses on health promotion strategies defocusing on weight, weight loss, and “healthy weight” (based on BMI); and (4) responds to the need of raising awareness of weight stigma and increasing the global evidence base [80]. It is our aspiration that this pilot intervention program may serve to develop a more effective protocol to reduce weight stigma and related aspects, ultimately improving the psychological and social well-being of adolescents.

## Figures and Tables

**Table 1:** Means (M) and standard errors (SE) by experimental condition and time.

Variable	Time 1		Time 2	
	MIC Group *(n* = 209) M (SE)	Intervention Group (*n* = 330) M (SE)	MIC Group (*n* = 209) M (SE)	Intervention Group (*n* = 330) M (SE)
WBIS	45.043 (2.988)	53.140 (2.027)	41.870 (3.271)	49.020 (2.218)
DEQ	27.751 (1.702)	30.110 (1.359)	28.276 (1.703)	30.407 (1.360)
ATOP	63.236 (1.006)	63.036 (0.796)	63.115 (1.042)	63.236 (0.824)
CDRS	−0.822 (0.132)	−0.875 (0.106)	−0.908 (0.127)	−0.787 (0.102)

Notes. DEQ: Disordered Eating Questionnaire; WBIS: Weight Bias Internalization Scale; ATOP: Attitudes Toward Obese Persons; CDRS: Contour Drawing Rating Scale.

**Table 2:** Bivariate correlations among variables measured at baseline (T1, *n* = 539).

	DEQ	WBIS	ATOP	SRS
DEQ	1	-	-	-
WBIS	0.700 *	1	-	-
ATOP	0.016	0.021	1	
CDRS	−0.610 *	−0.373 *	−0.015	1

Note. DEQ: Disordered Eating Questionnaire; WBIS: Weight Bias Internalization Scale; ATOP: Attitudes Toward Obese Persons; CDRS: Contour Drawing Rating Scale. * *p* < 0.001.

## Data Availability

The data presented in this study are available on request from the corresponding author due to the privacy and confidentiality of participants.

## Supplementary Materials

The following supporting information can be downloaded at: https://www.mdpi.com/article/10.3390/children12091208/s1, Table S1: Detailed results from two-way mixed ANOVAs. Note: * *p* < 0.05; † *p* < 0.10. Abbreviations: DEQ: Disordered Eating Questionnaire; WBIS: Weight Bias Internalization Scale; ATOP: Attitudes Toward Obese Persons; CDRS: Contour Drawing Rating Scale; SE: standard error.
