# Supplementary material for: A School-Based Intervention Program to Reduce Weight Stigma in Adolescents
Source: Children (Basel). 2025 Sep 10;12(9):1208. doi: 10.3390/children12091208 (PMC12468586; doi:10.3390/children12091208)
Supplement: Supplementary file 1 [file children-12-01208-s001.zip › children-3790649-supplementary.pdf]

	MIC	Intervention	Moment		Group		Moment*Group	
Variable	Mean (SE)	Mean (SE)	F value (df)	η_p^2	F value (df)	η_p^2	F value (df)	η_p^2
**WBIS**			7.518 (1,71)*	0.096	4.627 (1,71)*	0.061	0.126 (1,71)	0.002
T1	45.043 (2.988)	53.140 (2.027)						
T2	41.870 (3.271)	49.020 (2.218)						
**DEQ**			0.324 (1,473)	0.001	1.193 (1,473)	0.003	0.025 (1,473)	0.000
T1	27.751 (1.702)	30.110 (1.359)						
T2	28.276 (1.703)	30.407 (1.360)						
**ATOP**			0.005 (1,494)	0.000	0.001 (1,494)	0.000	0.076 (1,494)	0.000
T1	63.236 (1.006)	63.036 (0.796)						
T2	63.115 (1.042)	63.236 (0.824)						
**CDRS**			0.001 (1,470)	0.000	0.046 (1,470)	0.000	3.152 (1,470)†	0.007
T1	-0.822 (0.132)	-0.875 (0.106)						
T2	-0.908 (0.127)	-0.787 (0.102)						

Supplementary Table S1. Detailed results from two-way mixed ANOVAs. Note: *$p < .05$; $^{\dagger}p < .10$. Abbreviations: DEQ: Disordered Eating Questionnaire; WBIS: Weight Bias Internalization Scale; ATOP: Attitudes Toward Obese Persons; CDRS: Contour Drawing Rating Scale; SE: standard error.
